# A Blossoming Field of Research: How Florigen Is Transported to Create Flowers

**DOI:** 10.1371/journal.pbio.1001311

**Published:** 2012-04-17

**Authors:** Charles Q. Choi

**Affiliations:** Freelance Science Writer, New York, New York, United States of America

**Figure pbio-1001311-g001:**
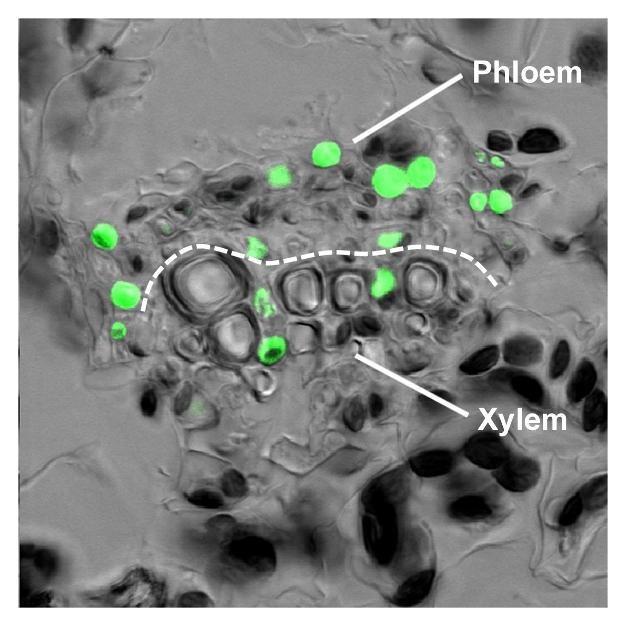
Overexpression of *FTIP1* deregulates the transport of FT:GFP protein from phloem to xylem.

[Fig pbio-1001311-g001]The act of flowering is the defining and most dramatic change that flowering plants undergo, and the fruits of such labor are crops that the world depends on. Scientists have long thought that the signal that switches the growing tip of a plant's shoot from making leaves to making flowers travels from the leaves to the shoot, but very little was known about how this signal made its voyage. Now Hao Yu at the National University of Singapore and his colleagues have provided the first insight into how this happens, by identifying a protein that is essential for this signal's movement.

Flowering is triggered by outside signals such as day length. Pioneering experiments revealed that plants used their leaves to perceive day length, sending a mystery signal, dubbed “florigen” in the 1930s, to a shoot's growing tip, formally known as its apical meristem.

After decades of effort, in 2007, a protein called FT was finally identified as part of the long-sought florigen. Although research did show that FT moved through the phloem—the vessels that carry sugars, amino acids, and other molecules created in the leaves elsewhere around the plant—it was completely unknown how it got transported. It seemed unlikely that simple diffusion could transport FT from leaves across the phloem all the way to the shoot, but it remained a mystery as to what proteins might help it on its way.

To find out, Yu and his colleagues scanned for proteins that interacted with FT using yeast two-hybrid screening. Basically, the FT gene was modified so that it produced FT and half of a transcription factor, a protein that binds to specific DNA sequences to influence genetic activity, in this case that of the *HIS3* and *lacZ* genes. Another gene was also modified in that yeast so it produced the other half of the transcription factor. If *HIS3* and *lacZ* got activated, that meant both halves of the transcription factor had come together, suggesting FT interacted with whatever protein the other gene created.

After scanning 3 million such yeast samples, the researchers identified a molecule they dubbed FT-INTERACTING PROTEIN 1 (FTIP1), whose messenger RNA (mRNA) expression patterns are similar to those of FT. Plants with mutant, non-functional versions of the *FTIP1* gene flowered much later in response to long days, and when such mutants were given a working version of the gene, their flowering time was restored largely back to normal. These findings suggested FTIP1 was key to how flowering was controlled over time.

To see where FTIP1 was active in plants, the researchers linked it with an enzyme known as beta-glucuronidase, or GUS, which stained blue when it reacted with an otherwise colorless compound, and found FTIP1 was active in the phloem. The main components of this plant tissue are sieve elements and companion cells. Sieve elements have pores in their cell walls, creating connections between vertically stacked cells, making them act like tubes. Companion cells move sugars and other compounds from leaves in and out of sieve elements, where they then travel around the plant. GUS staining revealed FTIP1 was located in companion cells.

By linking FTIP1 to green fluorescent protein (GFP), the scientists found FTIP1 was located in the endoplasmic reticulum (ER), which helps transport proteins in cells. Immunoelectron microscopy further revealed it was located in companion cells and in the channels linking companion cells and sieve elements, connections known as plasmodesmata that the ER membrane runs through.

To see if FTIP1 and FT interact, the researchers used in situ proximity ligation assays, which target a potentially interacting pair of proteins with pairs of antibodies that generate detectable circular DNA strands as markers if the interaction takes place. This revealed that FTIP1 and FT are often found in close proximity in vivo in companion cells.

When the scientists tagged FT with GFP, they saw it move to the shoot's growing tip in normal plants but not in ones that lost FTIP1 function. Immunoelectron microscopy further revealed that without FTIP1, FT accumulates in companion cells, with only very limited flow into sieve elements. These observations suggested FTIP1 regulated the export of FT from companion cells to sieve elements and thus to the shoot.

Although these findings suggest FTIP1 is key to florigen transport, other factors are likely to be involved. For example, although FT flow from companion cells into sieve elements is significantly compromised without FTIP1, it is not completely abrogated. This suggests that FT transport may also depend on other molecules, perhaps FTIP1-like proteins, or that compounds other than FT help to make up florigen. Potential candidates include the aptly named TWIN SISTER OF FT (TSF), a protein very similar to FT. TSF's expression patterns overlap with those of FTIP1, and it could be another molecule that FTIP1 influences the movement of.

Another mystery regarding FT is the precise route it travels through plasmodesmata in order to enter sieve elements—for instance, whether it travels within the ER or floats in the cytoplasm. Analyzing how FTIP is oriented in plasmodesmata could help to answer this question, Yu and his colleagues note.

Future work on florigen transport could also focus on homologs of FTIP1, of which there are 16 in the model plant *Arabidopsis* alone. The researchers' preliminary data hint that some of these homologs are involved in a wide range of plant developmental processes. This group of proteins may ultimately prove to be general regulators of macromolecules in plants, they suggest.

**Liu L, Liu C, Hou X, Xi W, Shen L, et al. (2012) FTIP1 Is an Essential Regulator Required for Florigen Transport. doi:10.1371/journal.pbio.1001313**

